# Improved Melting of Latent Heat Storage Using Fin Arrays with Non-Uniform Dimensions and Distinct Patterns

**DOI:** 10.3390/nano12030403

**Published:** 2022-01-26

**Authors:** Farqad T. Najim, Hayder I. Mohammed, Hussein M. Taqi Al-Najjar, Lakshmi Thangavelu, Mustafa Z. Mahmoud, Jasim M. Mahdi, Mohammadreza Ebrahimnataj Tiji, Wahiba Yaïci, Pouyan Talebizadehsardari

**Affiliations:** 1 Electrical Engineering Department, College of Engineering, Al-Iraqia University, Baghdad 10071, Iraq; farqad_alani@yahoo.com; 2 Department of Physics, College of Education, University of Garmian, Kalar 46021, Iraq; hayder.i.mohammad@garmian.edu.krd; 3 Department of Energy Engineering, University of Baghdad, Baghdad 10071, Iraq; hussein.alnajjar@coeng.uobaghdad.edu.iq (H.M.T.A.-N.); jasim@siu.edu (J.M.M.); 4 Center for Transdisciplinary Research, Department of Pharmacology, Saveetha Institute of Medical and Technical Science, Saveetha Dental College, Saveetha University, Chennai 600001, India; Lakshmi@saveetha.com; 5 Department of Radiology and Medical Imaging, College of Applied Medical Sciences, Prince Sattam bin Abdulaziz University, Al-Kharj 11942, Saudi Arabia; m.alhassen@psau.edu.sa; 6 Faculty of Health, University of Canberra, Canberra, ACT 2600, Australia; 7 Department of Mechanical Engineering, Qom University of Technology, Qom 3718146645, Iran; ebrahimnataj.m@qut.ac.ir; 8 CanmetENERGY Research Centre, Natural Resources Canada, Ottawa, ON K1A 1M1, Canada; 9 Centre for Sustainable Energy Use in Food Chains, Institute of Energy Futures, Brunel University London, Kingston Lane, Uxbridge, Middlesex UB8 3PH, UK

**Keywords:** latent heat storage, phase change materials, melting, triple pipe, fin arrays

## Abstract

Employing phase-change materials (PCM) is considered a very efficient and cost-effective option for addressing the mismatch between the energy supply and the demand. The high storage density, little temperature degradation, and ease of material processing register the PCM as a key candidate for the thermal energy storage system. However, the sluggish response rates during their melting and solidification processes limit their applications and consequently require the inclusion of heat transfer enhancers. This research aims to investigate the potential enhancement of circular fins on intensifying the PCM thermal response in a vertical triple-tube casing. Fin arrays of non-uniform dimensions and distinct distribution patterns were designed and investigated to determine the impact of modifying the fin geometric characteristics and distribution patterns in various spatial zones of the heat exchanger. Parametric analysis on the various fin structures under consideration was carried out to determine the most optimal fin structure from the perspective of the transient melting evolution and heat storage rates while maintaining the same design limitations of fin material and volume usage. The results revealed that changing the fin dimensions with the heat-flow direction results in a faster charging rate, a higher storage rate, and a more uniform temperature distribution when compared to a uniform fin size. The time required to fully charge the storage system (fully melting of the PCM) was found to be reduced by up to 10.4%, and the heat storage rate can be improved by up to 9.3% compared to the reference case of uniform fin sizes within the same fin volume limitations.

## 1. Introduction

The transition from fossil fuels toward renewable energies such as sunlight and wind is widely recognized these days as the most important step toward converting the global energy system into one that is both economically and environmentally sustainable. Due to the fact that almost all sources of renewable energy are fluctuating and intermittent in their availability and amount, energy storage is essential for the widespread adoption of renewable energy technologies. Thermal energy storage (TES) is one of the most promising energy storage technologies available today, and it is also one of the most cost-effective options [1]. It is being advocated because it has the potential to provide consistent production of power from these sources, preserving the limited fossil-fuel sources, and lowering the need for pricey natural gas and oil imports [2]. Energy can be supplied on a diurnal or seasonal basis by TES technologies, resolving any potential imbalance between energy in supply and use. So, as a consequence, developing efficient designs is very important to carefully match the suitable TES technology to each specific application of renewable energy.

TES is a term that refers to the temporary holding of energy in thermal form for future utilization. It can be classified into three categories according to the materials used. Sensible TES by raising or lowering the temperature of the material via altering its sensible heat, latent TES by changing the phase of the material by alerting its latent heat, and thermochemical TES by storing or releasing heat by cyclic endothermic and exothermic chemical reactions. The latent TES, which makes use of what are known as phase-changing materials (PCMs), is favored above the others for two reasons. First, the latent TES systems based on PCMs are far more compact than sensible TES. For instance, the volume percentage of latent TES to that of rock-based TES is around 1 to 17 [3]. Second, the thermal properties of phase transitions in PCMs make allowances for only a little or no temperature degradation, which means that the temperature may stay almost constant throughout the operating duration. Therefore, PCMs find a wide range of applications such as building energy management [4], load control in heating and cooling utilities [5,6], and peak shaving in renewable energy plants [7,8,9,10,11,12,13,14,15].

Material thermodynamic properties such as thermal diffusivity, point of phase transition, and latent heat of fusion per unit mass all contribute significantly to the efficacy of PCM as a useful storage substance. Nevertheless, the most significant inconsistency that almost all PCMs today suffer from is their intrinsically low heat conduction, which has a negative impact on the system’s thermal reaction to the cyclic heat charging/discharging operations. To overcome this issue, researchers identified different approaches for the thermal enhancement of TES systems, such as porous matrices [16,17,18,19], extended fins [20,21,22,23,24,25], and heat pipes [26,27] along with utilizing good performing casing for PCM containment. Boosting the thermal performance of PCM-based storage devices by applying extended fins to the tubes transporting the heat-transfer fluid (HTF) is often considered as one of the most effective enhancement methods in energy storage systems [23,28]. When fins are fabricated and installed appropriately, they may achieve a high enhancement ratio, save the material used, and enhance the system’s compactness. Therefore, optimizing the design, size, arrangement, and material use of fins has become a major area of interest within the field of thermal energy systems [29,30]. The optimization of these parameters is carried out depending on the type and features of the TES technique, the kind of PCM utilized in the application, and the targeted heat charging/discharging rates.

The useful application of extended fins of longitudinal, circular, angular, tree-like, and twist-shaped configurations has been a topic of interest to several studies over the last three decades. The potential for melting enhancement of PCM in a shell-and-tube casing was studied by Lacroix [31] who revealed that the introduction of circular fins is more influential at lower intake temperatures (ΔT ≥ 5 K) and moderate HTF flow rates (m ≤ 0.015 kg/s). Effectiveness of the V-shaped longitudinal fin in cylindrical storage assemblies with paraffin RT60 as PCM was investigated by Velraj et al. [32], and it was determined that the time required for complete solidification is approximately reduced by (1/number of fins) when compared to the case of no fins. Gharebaghi and Sezai [33] compared the performance of a rectangular TES unit equipped with vertical fins on the thermally active horizontal wall to that of the same unit with horizontal fins on the thermally active vertical wall and found that the latter was more efficient in terms of the heat-transfer enhancement rate. Al-Abidi et al. [34] examined the impact of including longitudinal fins on PCM charging response in the triple-tube TES unit under various HTF temperature and flow conditions, and the results indicated that the HTF temperature has a greater effect on the enhanced melting rate than the HTF flow rate. Sciacovelli et al. [35] achieved a 24% higher discharging efficiency with the application of Y-shaped fins in the PCM-based shell-and-tube storage system, and the results indicated that the short-duration operation of PCM systems requires larger Y-shaped fin angles, whereas the longer duration operation requires smaller Y-shaped fin angles.

Abdulateef et al. [36] compared the melting enhancement of PCM in horizontal triplex-tube units with longitudinal and triangular fins and it was reported that a 15% faster melting rate can be achieved with the application of triangular fins compared to that of longitudinal fins. As the melting rate is not the same at the different parts of the TES units, Mahdi et al. [24,37] suggested employing fewer and smaller fins at the top part with longer fins at the bottom part of horizontal TES units to further support the enhancement potential of longitudinal fins during the heat storage mode. To maximize the contribution of natural convection during melting in vertical TES units, Singh et al. [38] suggested the use of non-uniform distribution of annular fins with a progressive drop in the fin height. To further enhance the PCM charging response, Ghalambaz et al. [39] introduced twisted fins as innovative TES enhancer and reported 42% saving in melting time with a 63% increase in heat storage rate with the inclusion of twisted fins compared to that of longitudinal fins within the same PCM mass limitations. Further, the PCM has been used as a promising technique for the thermal management of the car’s battery [40,41].

Based on the literature survey above, there are still several fin parameters that require further investigations to reveal their role as a good performing enhancer in the design of PCM-based systems with expanded fins. The influence of reducing the circular fin’s size (length and thickness) in one system was never considered in the literature. This work investigates the novel design of the thermal energy storage system, which involves the incorporation of various fin sizes into the same system. Additionally, combining a flat fin at the bottom part of the geometry is also being examined to evaluate the expedited melting process for the solid PCM parts being collected at the base of the system. The geometric parameters of the fin array, such as their size, placement, and arrangement, have a significant effect on the buoyancy-driven flow of liquid PCM and the overall thermal effectiveness of the storage system. Therefore, a simulation model for the PCM melting process in a vertical triple-tube casing with circular fins was developed and implemented to identify the impact of modifying the fin geometric characteristics and distribution patterns in various spatial zones of the heat exchanger. The primary objective was to optimize the size, arrangement, and vertical placement of the fin array during the charging phase of PCM under a variety of temperature and flow conditions. The results indicated that the optimal structure of the fin system depends on the dimensions and distribution patterns of the fin arrays. Finally, three additional cases were compared: the optimum case with uniform fin dimensions, the optimum case with nonuniform fin dimensions, and the reference case with no fins to reveal the superior effectiveness of the proposed fin system.

## 2. Problem Description

**Figure 1:**
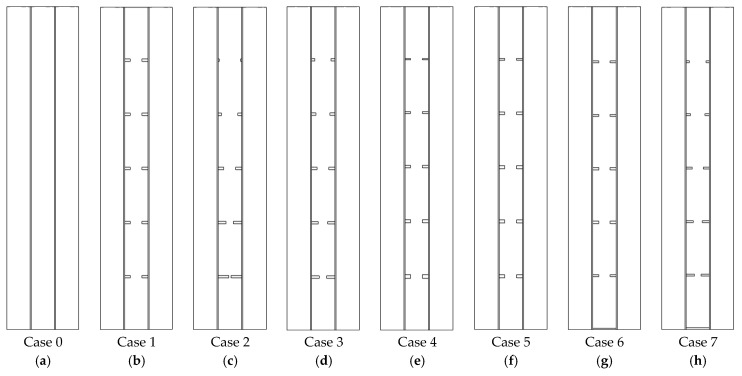
The Two-dimensional structure of the studied vertical triple-tube heat exchanger cases including (**a**) case 0 (finless case), (**b**) case 1 (uniformly distributed case), (**c**) case 2 (the fin’s height is changed from 9 mm to 1 mm), (**d**) case 3 (the fin’s height in case 3 is changed from 7 mm to 2 mm), (**e**) case 4 (the thickness of fins is changed from 3 mm to 1 mm), (**f**) case 5 (the thickness changed from 2.5 mm to 1.5 mm), (**g**) case 6 (flat fin integrated to the bottom of case 1), and (**h**) case 7 (flat fin integrated to the bottom of case 3).

A heat-exchanger in the form of a triple-pipe PCM casing with circular fins is investigated in this study. The interior and exterior pipes are peripherally finned with a total number of 10 fins. The five fins with non-uniform dimensions are linked to the interior pipe and the other five are attached to the exterior pipe of the PCM casing. The PCM occupies the space inside the middle pipe while water as the heat-transferring fluid (HTF) is circulated through the interior and exterior pipes. The proposed system is displayed in Figure 1. The system with fins of non-uniform dimensions is assessed with reference to the case of uniform fins (case 1) and no-fin (case 0). In the system with uniform fins, the dimensions of all fins are similar equal to 2 mm × 5 mm. For the finned cases with non-uniform dimensions, first, the height of the fins is changed in cases 2 and 3 considering the constant fin’s thickness equal to 2 mm. In case 2, the fin’s height is changed from 9 mm to 1 mm while the fin’s height in case 3 is changed from 7 mm to 2 mm. In cases 4 and 5, the heights of the fins are constant equal to 5 mm; however, the thickness of fins is changed from 3 mm to 1 mm in case 4 and from 2.5 mm to 1.5 mm in case 5.

In vertical heat storage systems and owing to the existence of natural convection phenomenon during the melting phase, the PCM at the upper section of the storage system is melted at a higher rate than the PCM at the bottom [37]. Thus, in this study, as a distinct pattern for the first time, to boost the storage performance of the system during the heat charging mode, an integrated fin in cases 6 and 7 is placed at the bottom of the heat exchanger. In case 6, in addition to the added fin at the bottom, uniform dimensions are considered for the other fins while in case 7, a fin is added to the bottom wall of the heat exchanger for the best-proposed system among cases 2–5 (this is studied later in the discussion section and is achieved as case 3). The fins’ dimensions related to the studied cases are presented in Table 1.

**Figure 2:**
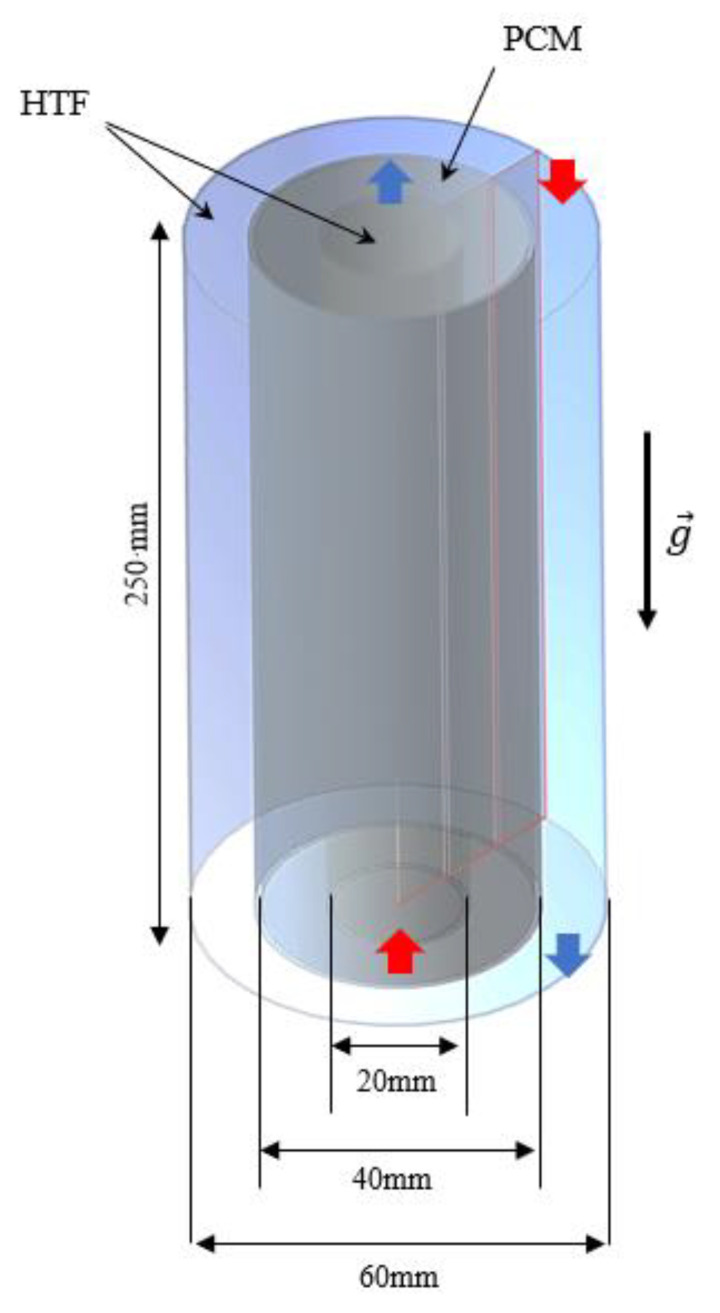
The schematic of the studied vertical triple-tube heat exchanger including the boundary conditions and dimensions for the no-fin case.

It should be noted that the system’s length is 250 mm and that the diameters of the interior, middle and exterior pipes are 20, 40, and 60 mm, respectively. The pipe walls, which are made of copper, are equally fixed as 1-mm wide. The HTF is circulated in the inner pipe in the opposite direction of gravity, while it is circulated in the gravity direction inside of the outer pipe. As established in the literature [42,43], such a configuration provides superior performance when compared to co-current directions for the fluid flow. As a result, the flow of HTF through the PCM casing is kept as counter-current. The conditions for the HTF at the inlet section are constant velocity and temperature, whereas the boundary conditions for the HTF at the outlet section are outflow with constant velocity and temperature, respectively. A 3D representation of the PCM heat-exchanger casing with no fins is presented in Figure 2 along with the dimensions of constituent pipes and their associated boundary conditions. The flow is regarded as axisymmetric due to the type and features of the studied problem and the absence of circumferential flow variation in the system under investigation (shown in Figure 2). Values of 50 °C inlet temperature and 1000-Reynolds number are used for the HTF flow to establish the optimal fin arrangement. The value 15 °C is considered as the initial temperature for heating of PCM during the heat charging mode.

The PCM in use is paraffin RT-35, whose thermophysical characteristics are reported in Table 2.

## 3. Mathematical Modeling

To numerically simulate the phase transition of PCM in use, the enthalpy–porosity approach was implemented [45,46]. In this approach, the liquid fraction and the porosity were assumed to be equivalent inside all cells of the computational domain. To drive the governing equations, the following assumptions are made [47,48]:

- Applying the Boussinesq approximation to figure out the density and buoyant force variations.
- Assuming the flow of liquid PCM is transient, axisymmetric, laminar, and incompressible.
- Taking acceleration of gravity is along the negative y-axis.
- Neglecting heat loss into the surroundings due to the good thermal insulation at the exterior boundaries.
- Applying no velocity-slip boundary conditions at the solid boundaries.

On the next section are presented the Navier-Stokes conservation equations for continuity, momentum, and energy [49]:

(1) $$\frac{\partial \rho }{\partial t}+\nabla \cdot \rho \vec{V}=0$$

(2) $$\rho \frac{\partial \vec{V}}{\partial t}+\rho \left(\vec{V}\cdot \nabla \right)\vec{V}=-\nabla P+\mu \left(\nabla^{2}\vec{V}\right)-\rho_{ref}\beta \left(T-T_{ref}\right)\vec{g}-\vec{S}$$

(3) $$\frac{\rho C_{p}\partial T}{\partial t}+\nabla \left(\rho C_{p}\vec{V}T\right)=\nabla \left(k\nabla T\right)-S_{L}$$

The last term ($\vec{S})$ in the second conservation equation is included to account for the influence of phase transition, which is specified as the velocity damping component of the Darcy law [50]:

(4) $$\vec{S}=A_{m}\frac{{\left(1-\lambda \right)}^{2}}{\lambda^{3}+0.001}\vec{V}$$

where the mushy zone constant $A_{m}$ is considered 10^5^ according to the literature [29]. It would be worthy to mention that there is a semi-liquid zone exists between the melted and solidified zones of PCM during phase transition. So, high values of $\vec{V}$ indicate sharper transitions of material velocity to zero during solidification. This, in turn, affects heat transport and flow properties during melting and solidification as high readings of damping velocity may produce oscillations in the predicted numerical solution.

For the effect of latent heat and phase change process in the energy equation, a source term is added where λ (liquid fraction of PCM) is introduced as [41]:

(5) $$\lambda =\frac{\Delta H}{L_{f}}=\left\{\begin{array}{c} 0\;if\;T<T_{Solidus}\\ 1\;if\;T>T_{Liquidus}\\ \frac{T-T_{Solidus}}{T_{Liquidus}-T_{Solidus}}\;if\;T_{Solidus}<T<T_{Liquidus} \end{array}\right\}$$

The Boussinesq approximation is applied to compute the density fluctuations due to the temperature swings during the PCM’s phase transition course. In this approximation, fluid density is handled as constant, except in the gravity part of the momentum equation, where density is regarded as a temperature-dependent variable [16]:

(6) $$\rho =\rho_{ref}\left(1-\beta \left(T-T_{ref}\right)\right)$$

The source term $S_{L}$ in the third conservation equation is described as follows:

(7) $$S_{L}=\frac{\rho \partial \lambda L_{f}}{\partial t}+\rho \nabla \left(\vec{V}\lambda L_{f}\right)$$

The rate of energy stored during the melting phase is estimated as:

(8) $$\dot{E_{T}}=\frac{E_{e}-E_{i}}{t_{m}}$$

where $t_{m}$ is the melting time and $E_{e}$ and $E_{i}$ are the total heat storage in PCM upon ending and starting of the phase-transition course. E is the summation of sensible heat $\left(MC_{p}dT\right)$ and latent heat $\left(ML_{f}\right)$ of the PCM. The flow of the HTF flow is assumed laminar in the present analysis.

## 4. Numerical Model

**Figure 3:**
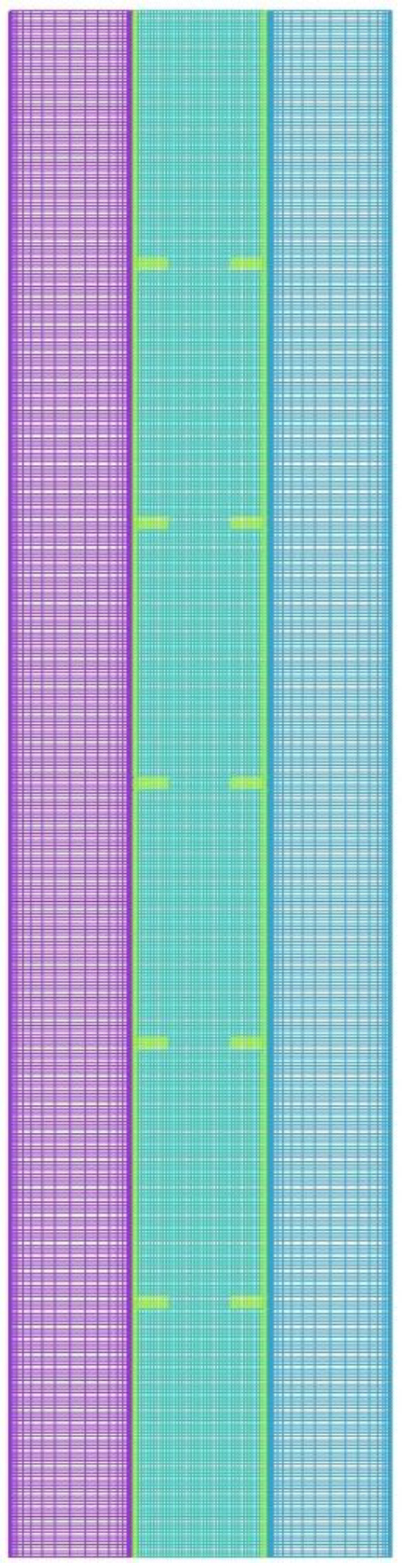
Structure of the mesh selected after the grid independence test.

A numerical simulation is a trustworthy approach for assessing the functioning of a given system design before fabrication so that any design modifications can be approved or rejected [51,52,53]. In this study, a modified ANSYS-FLUENT simulation solver based on SIMPLE model for pressure-velocity coupling and Green-Gauss meshing approach was used to examine the thermofluidic performance characteristics of PCM throughout the heat charging mode. The QUICK differencing approach was utilized to solve the momentum and energy equations, while the PRESTO scheme was employed to solve the pressure correction equations. Following a thorough pre-selection, the under-relaxation factors for pressure correction, velocity components, liquid fraction, and energy equation are set to 0.3, 0.3, 0.5, and 1, respectively. For the continuity, momentum, and energy equations, the convergence requirements for ending the iterative solution are set to 10–4, 10–4, and 10–6, respectively. The grid, as well as timestep size independence tests, are conducted. For this purpose, different cell numbers of 28,500, 43,000, and 81,620 are assessed using the timestep size of 0.2 s for the finned triple-pipe with uniform fins. Table 3 shows the melting time for different sizes of the grids in use. The outcomes, as shown in the table, are almost identical for the grid sizes of 43,000 and 81,620, and therefore, the mesh size of 43,000 is chosen for further analysis. It should be noted that a denser mesh is selected at the boundaries in both the PCM and HTF domains. Table 3 also presents the melting time for different sizes of time step size for the selected grid with 43,200 cells. As shown, the outcome data for the time step sizes of 0.1, 0.2, and 0.4 s investigated are nearly identical, particularly for the values of 0.2 and 0.1s. As a result, 0.2 s is adopted as the time step size in this research. The configuration of the selected mesh after the grid independence test is shown in Figure 3.

**Figure 4:**
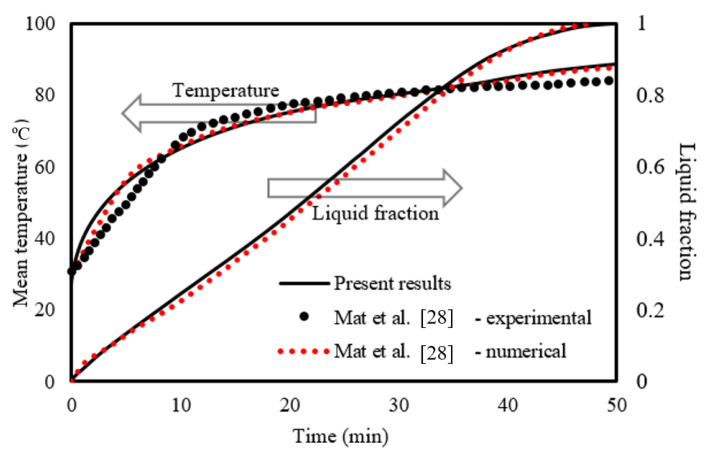
Comparison of the simulation model’s temperature and liquid-fraction evolution predictions to those of Mat et al. [28].

To validate the suitability of the simulation model developed, the findings of Mat et al. [34] were used as referential, and the geometry utilized in that investigation was regenerated. Mat et al. [34] numerically and experimentally evaluated a double-tube casing unit utilizing RT58 as the PCM. The study by Mat et al. was used as referential to validate the present study since the geometries examined in the two studies are almost similar. The referential study investigated the presence of inserted fins attached to both the interior and exterior tubes of the PCM shell in a staggered arrangement with the inner tube having a constant wall temperature. Two performance parameters were utilized to determine the validity of this work: the overall temperature of the PCM and the transient development of the liquid fraction. Figure 4 shows the results of the validation study, which indicate that the numerically predicted and experimentally measured data points of Mat et al. [34] are very equivalent to the present model predictions. The maximum error of the liquid fraction and the mean temperature for the numerical results for the current work against the work of Mat et al. [28] is found to be 1.5% and 0.78%, respectively. However, the error of the mean temperature of the current work and the experimental side of Mat et al. [28] is 4%.

## 5. Results and Discussion

This section studies the effects of the fins’ distribution design and the dimension on the charging process. The finned case with uniform fins is compared with the finless circumstances. Then different dimensions of the fins are explored, considering various lengths with a constant width and various widths with a constant length. Further, an additional fin was added for the bottom of cases 1 and 3 to enhance the thermal behavior of PCM during melting. All the studied cases were assessed by analyzing the temperature contour track record and the history of the liquid-fraction progression during the heat charging process.

### 5.1. Impact of Uniforms Addition Fin in the Compared with the No-Fin Case

Applying fins in the thermal energy storage system improves the thermal performance owing to increasing the surface area of the heat transfer and enhancing the mean thermal conductivity of the whole domain. The reason is that the thermal conductivity of the added fins is greater than that of the PCM. The fins guarantee the delivery of heat deeply in the region of the PCM domain, which improves the heat distribution in the PCM domain. Once the PCM melts, the thermal convection is affected more by the attendance of fins. This part of the study included a domain with five fins with a length of 5 mm and a thickness of 2 mm, in comparison with the case of no-fins.

**Figure 5:**
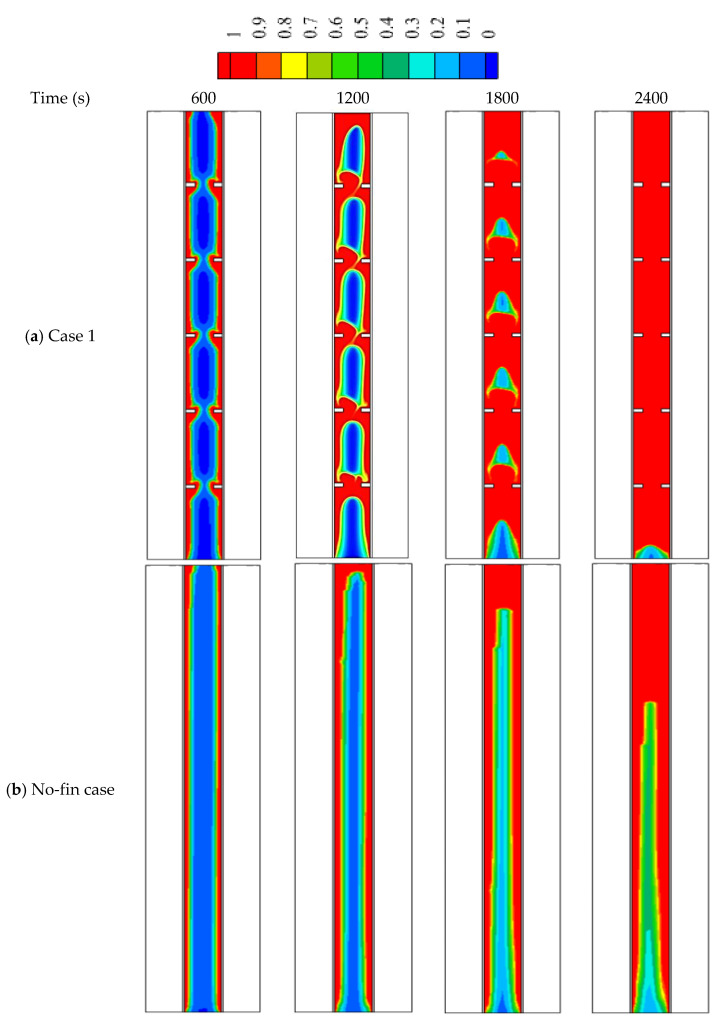
The liquid development in (**a**) case 1 (uniform size and inline fins) and (**b**) no-fin case at different time.

Figure 5 shows the development of the liquid fraction in the uniform distributed fins compared with the no-fins (fin-less) case. The figure shows that the melting process performs in the region besides the walls and around the fins. The solid phase in the regions amongst the fin regions is still connected at the time of 600 s. This connection is disappearing within 1200 s, because of the considerable among of transferred heat from inline fins. The solid part shrinks with time, and they held over the patch of the fins. Within 2400 s, only 2% of the solid part can be seen at the base of the system. For the no-fin case, the melting process takes a longer time to cover the same spaces compared to the inline fins case, due to the limited surface area caused by the absence of the fins. Within 2400 s, only 70% of the total PCM was melted.

**Figure 6:**
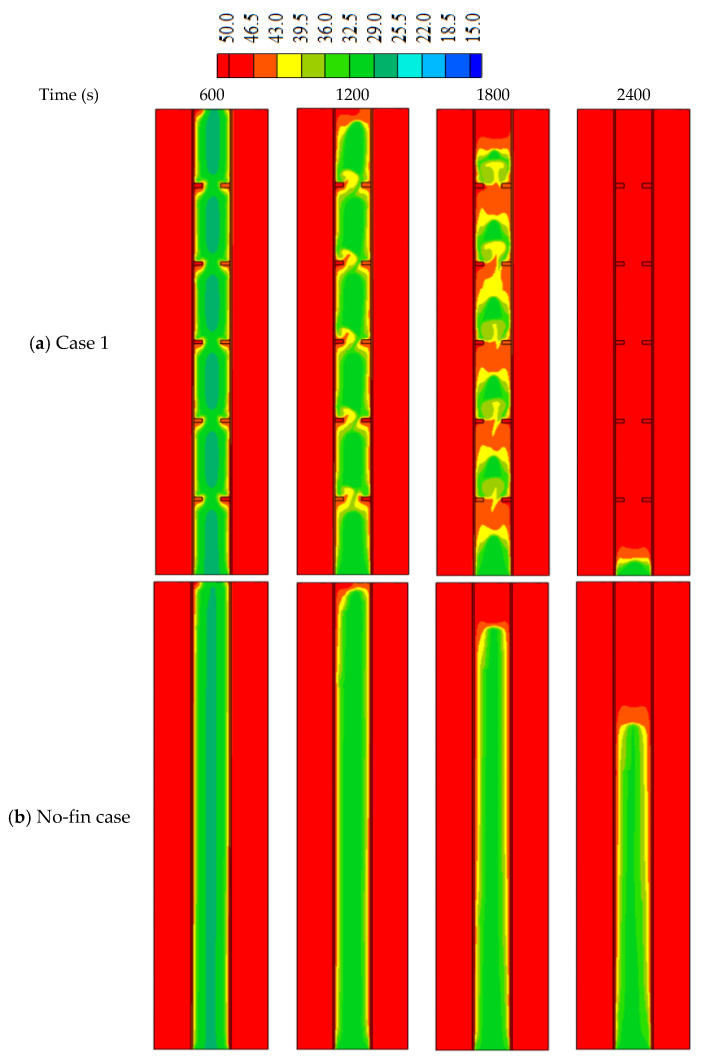
The temperature distribution in (**a**) case 1 (uniform size and inline fins) and (**b**) no-fin case at different time.

The temperature of HTF is stabilized at 50 °C, and the temperature of the PCM rises at the region close to the walls and around the fins, as shown in Figure 6. The average temperature of the PCM increases rapidly within the first 600 s due to the heat conductivity of the solid fins (before the melting process). The warm region gathers at the top part of the system. The average temperature of the PCM reaches 49 °C within 2400 s. This phenomenon is not detected in the fin-less case; in which the temperature increases slightly, as the heat transfers to the PCM through only that separated wall. The average temperature of the PCM increases to 40% within 2400 s.

**Figure 7:**
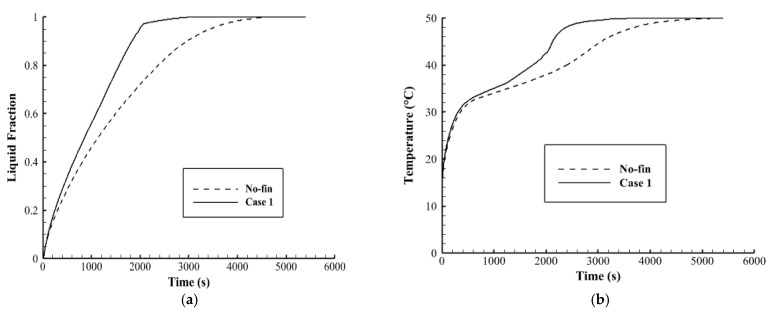
(**a**) liquid fraction, (**b**) average temperature profiles for case 1 and the fin-less case during the melting process.

Figure 7a illustrates the liquid fraction development till the fully molten process. The figure shows that in case 1, The melting process increases rapidly during the first 2000 s, because of the high heat transfer rate to the PCM through the fins as well as the effect of the thermal conduction. After 2000 s, the melting process goes with a slower mode because of the natural convection, which is caused by the converting of the most PCM to the liquid phase. For the finless case, the molten PCM growth logarithmically in slow modes because the heat transfers only due to the conduction effect. The converting process goes fast, then it slows down gradually because of the development of the natural convection. Figure 7b illustrates the mean temperature rising of the PCM in both case 1 and the fin-less case. The figure shows that the temperature increases sharply for both cases during the first 400 s due to the conduction process. After, the temperature growth goes slower due to the development of the convection effect, especially in the fin-less case. In case one, the effect of the fins reduces the impact of the Thermal convection. The total melting times are 3056 s and 4654 s for case 1 and the finless case, respectively, as shown in Table 4, which also indicates that the heat storage rate (the amount of the stored heat per unit time) in case 1 (55.1 W) is much higher than that in the finless case (36.2 W). It can be concluded that the thermal performance in case 1 is much more efficient than that in the fin-less case.

### 5.2. Impact of Non-Uniform Dimensions in Fin Arrays

In this part of the study, non-uniform dimensions of the fins are considered. In cases 2 and 3, the height of the fins is changed considering the constant value for the width as mentioned in the system discretion. In cases 4 and 5, the width of fins is non-uniform considering the constant value for the height.

**Figure 8:**
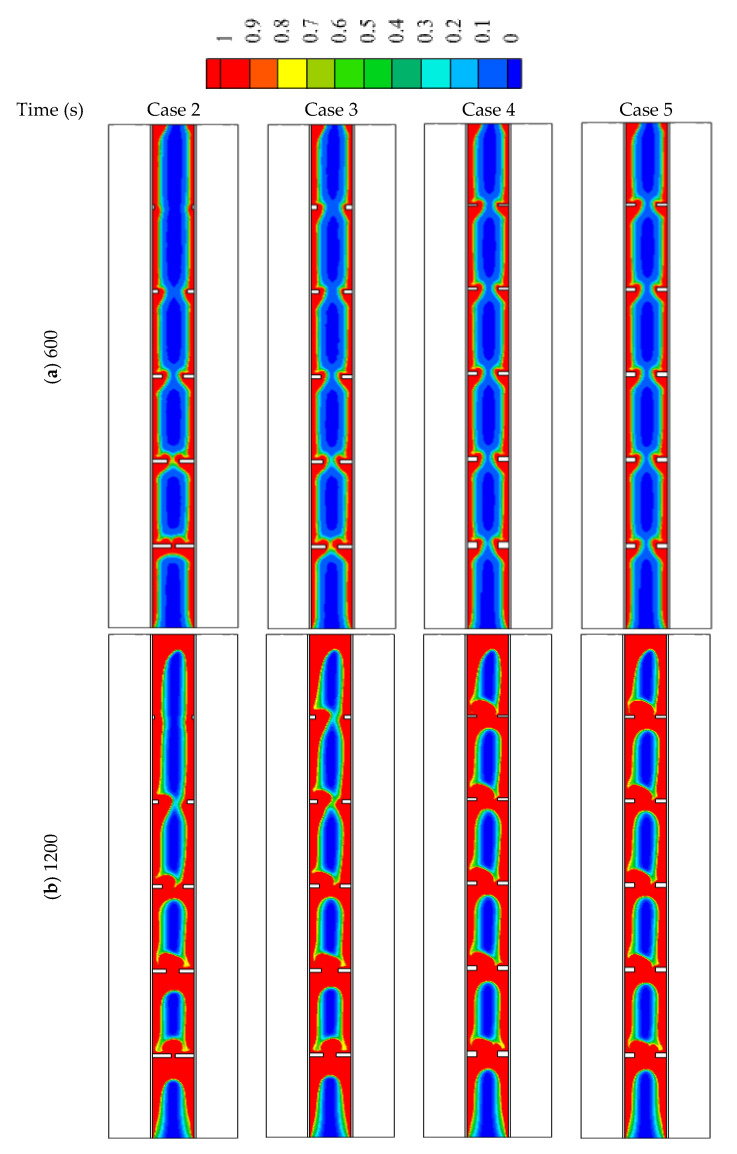

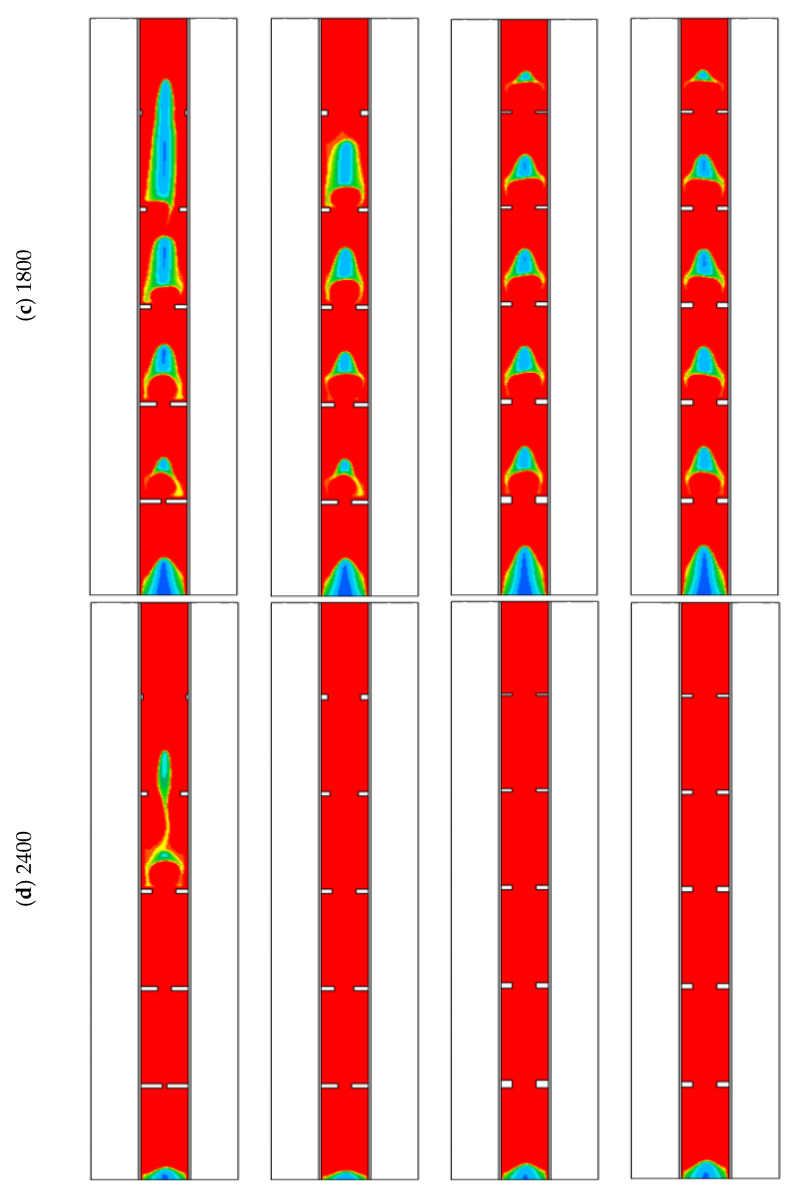
The liquid development in different cases of non-uniform sizes of the fins for the times of (**a**) 600, (**b**) 1200, (**c**) 1800 and (**d**) 2400.

Figure 8 illustrates the liquid fraction of the cases (2, 3, 4, and 5) during different time steps. For the first 600 s, the behaviors of the malting process look similar, as the conduction heat transfer dominates the process. The behavior slightly changed for the various cases because of the different surface areas of the fins. As the molten PCM covers the wall and the fins, the effect of the convection heat transfer appears clearly on the system. For case 2, the solid part extends on the areas over the highest fin (1 mm × 2 mm) at the time of the 1800 s due to the short length of that fin. For the other cases, the solid part is confined between the fins except for the highest part of case 2 which is molten completely. At 2400 s, most of the PCM melts in all the cases excluding the lowest part of the system due to the buoyancy effect, which helps to collect the solid part at the bottom and rise the liquid phase up due to the density differences.

**Figure 9:**
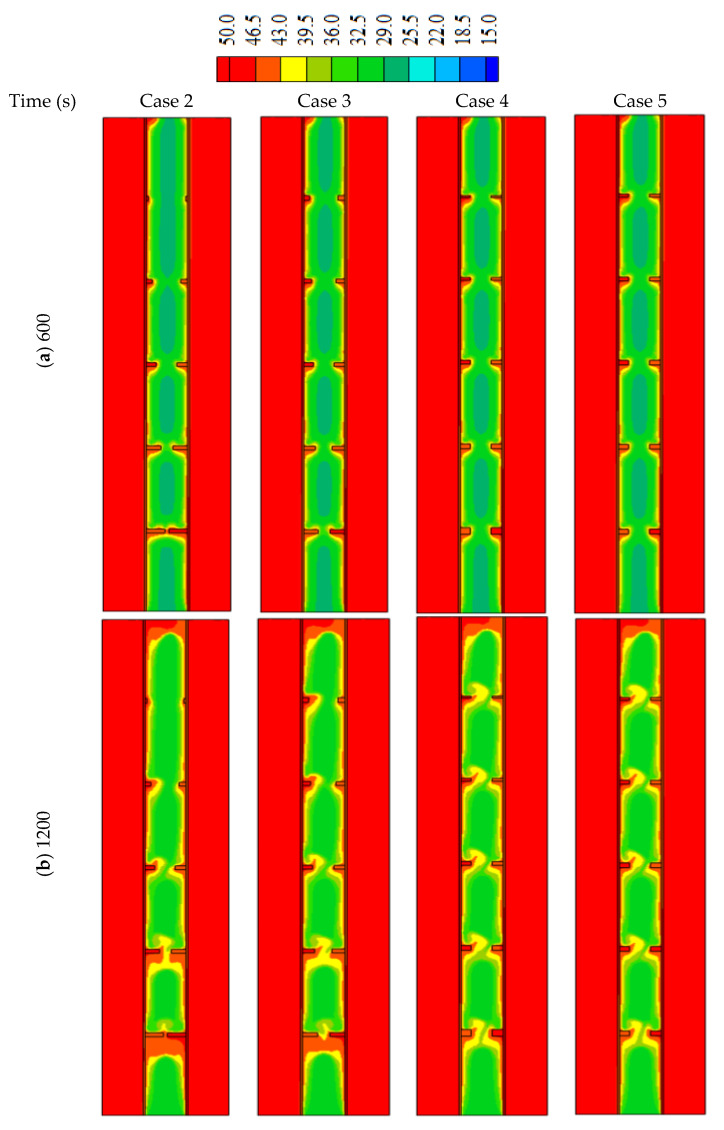

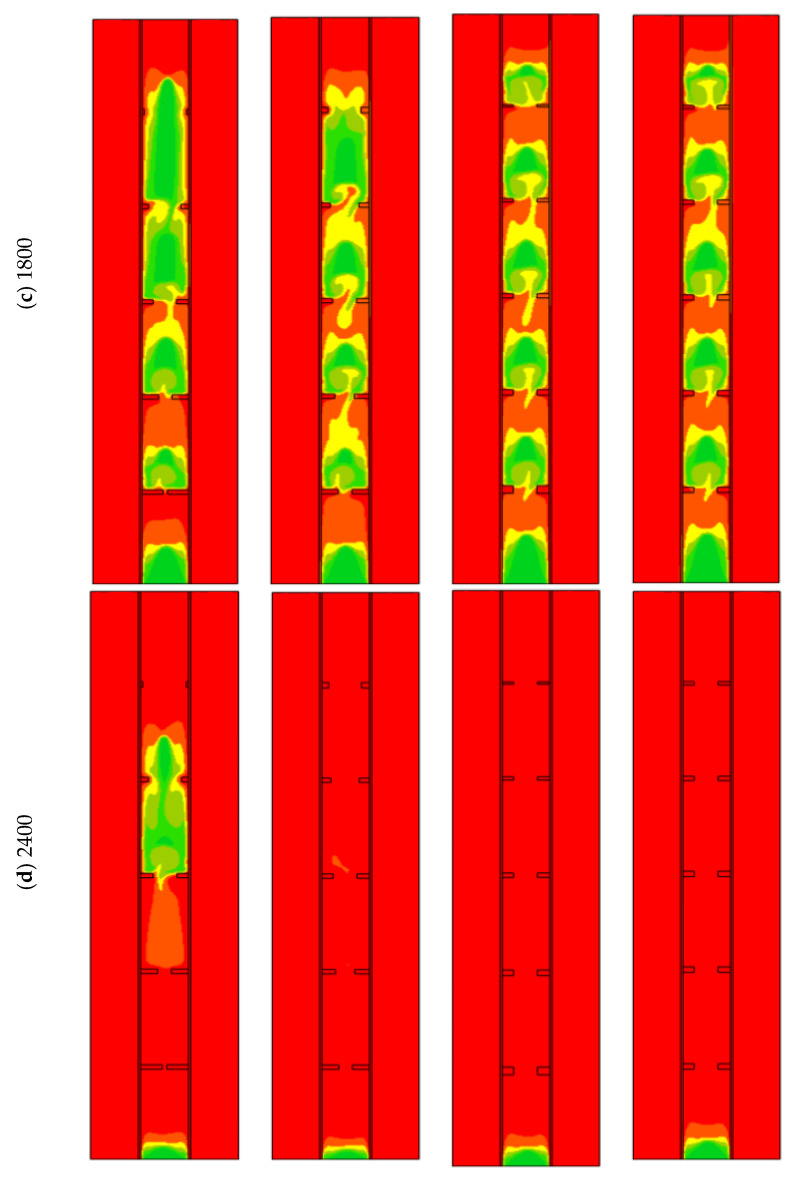
The temperature profiles in different cases of non-uniform sizes of the fins for the times of (**a**) 600, (**b**) 1200, (**c**) 1800 and (**d**) 2400.

**Figure 10:**
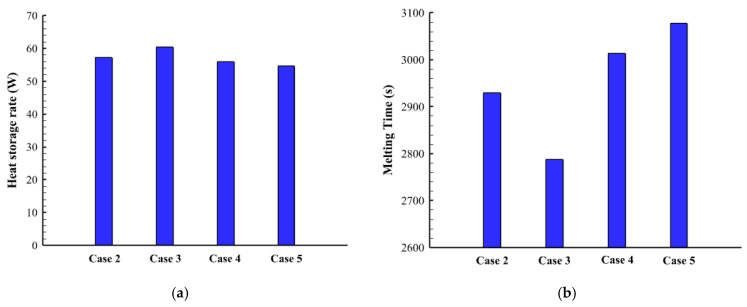
(**a**) Heat storage rate in Watt, (**b**) Melting time in seconds for different cases of the non-uniform fins sizes during the melting process.

Figure 9 shows the temperature profile of cases (2, 3, 4, and 5) at different time steps. Within 600 s, the average temperature for all the cases is almost the same, as the effect of the conduction is appearing in all the cases. The temperature rises around the fin and beside the wall and expands gradually to the deep part of the PCM. The top region of the system reaches the thermal equilibrium first due to the collection of the liquid PCM at the top of the region. All the regions in cases 3, 4, and 5 reach the thermal equilibrium except the bottom part. In case 2, there is a patch of a solid part, which appears colder because the fin around is smaller than the other, relatively. The heat storage rate shows a maximum value in case 3 and has also a minimum melting time as shown in Figure 10. This behavior is caused by the dimensions of the fins in case 3, which is longer in suitable size at the bottom (the place of collecting the solid phase) and becomes shorter as higher be raised. The maximum melting time with the lowest heat transfer rate is shown in case 5. The heat storage rate in all the cases is almost the same within a range between 54–60.5 W (Figure 6a) and the total melting time changes between the range of (2780 s–3077 s) (Figure 10b). Table 5 shows the values of the melting time and the heat storage rate for cases 2, 3, 4, and 5. Case 3 with the highest storage rate (16.32 W) has an advantage over cases 2, 4, and 5 by 5.2%, 7.4%, and 9.3%, respectively. However, the melting time of case 3 (2787 s) is shorter than cases 2, 4, and 5 by 5.1%, 8.1%, and 10.4.

### 5.3. Impact of Adding a Fin to the Bottom of the PCM Shell

In this part of the study, an extra fin is added for cases 1 (uniform fins dimension) and 3 (the best case with non-uniform fins dimensions) and they are compared with the original cases. Case 6 is the modified version of case 1 (after adding the fin at the base of the shell), and case 7 is the modified version of case 3. It should be noted that the total sizes of the fins are the same to grantee the same mass of the utilized PCM.

**Figure 11:**
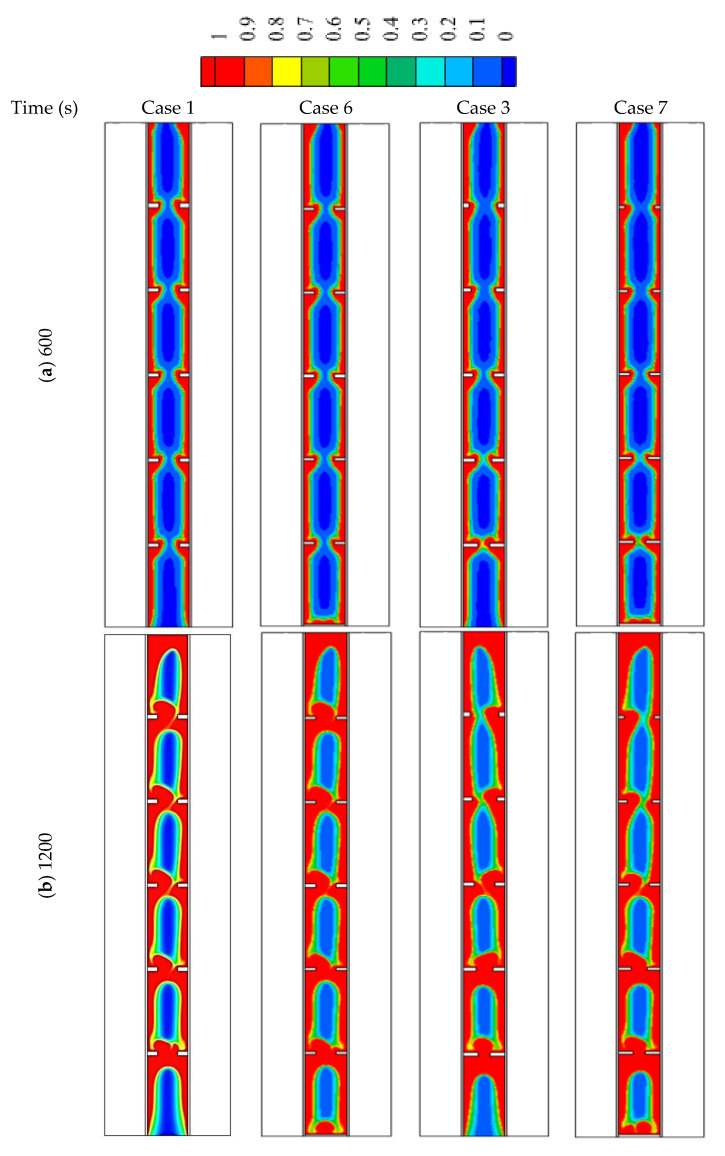

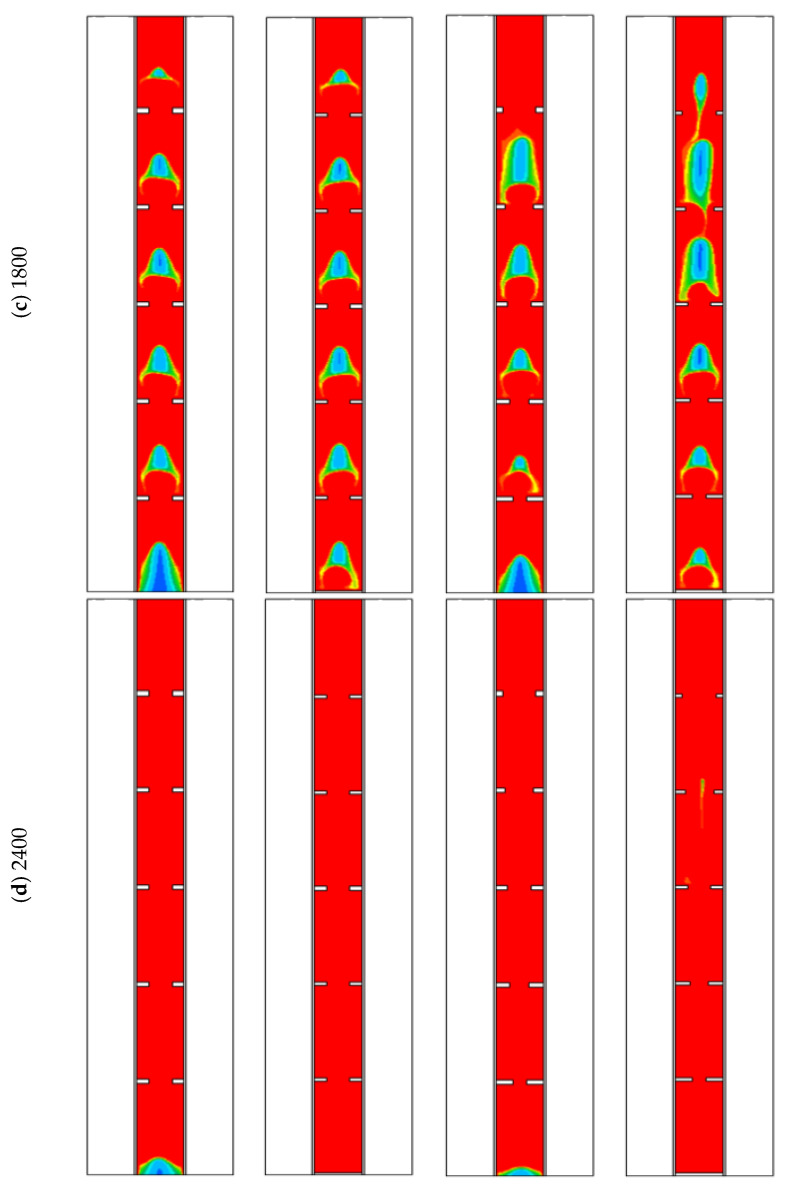
The liquid development in cases 1, 3, 6, and 7 for for the times of (**a**) 600, (**b**) 1200, (**c**) 1800 and (**d**) 2400.

**Figure 12:**
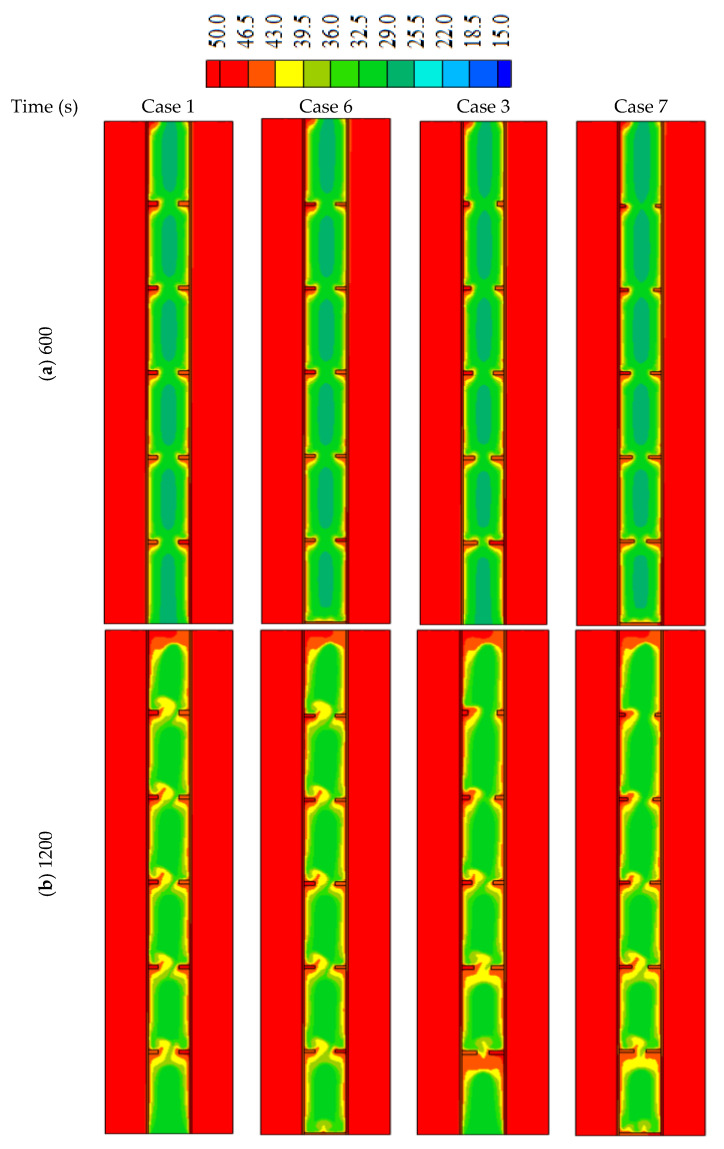

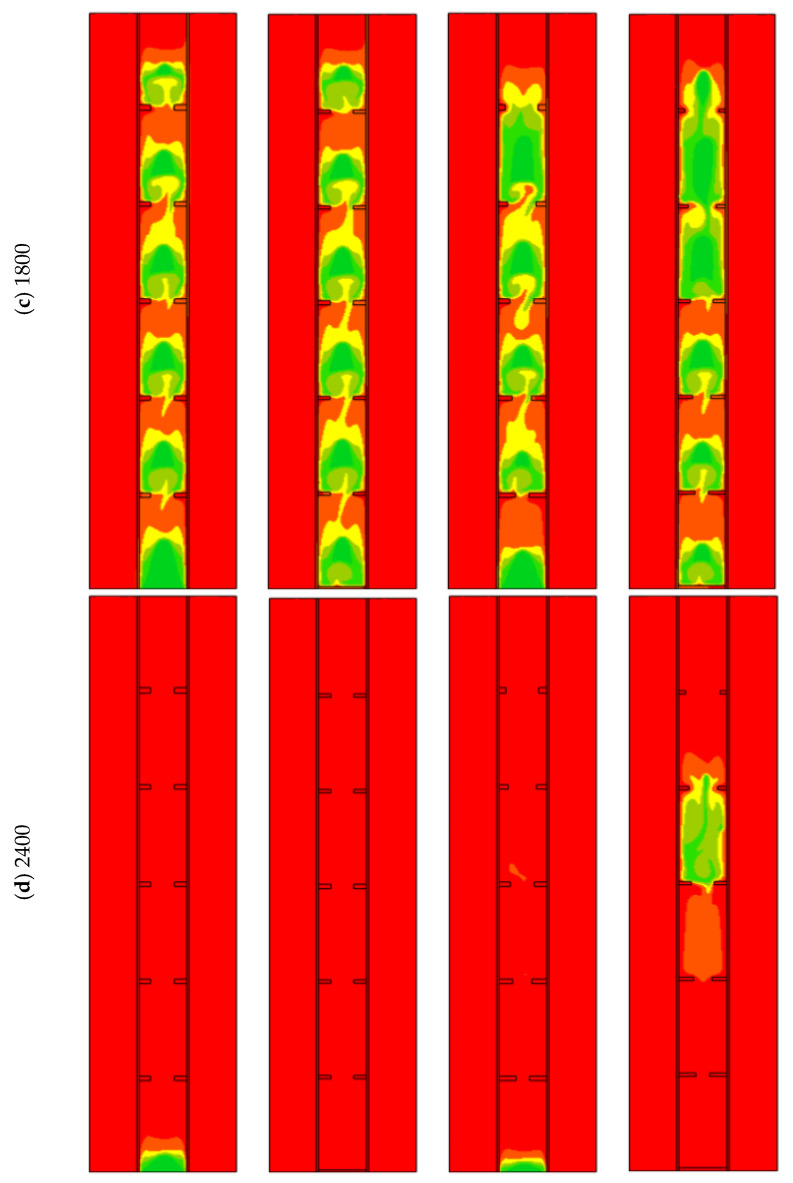
The temperature profiles in cases 1, 3, 6, and 7 for the times of (**a**) 600, (**b**) 1200, (**c**) 1800 and (**d**) 2400.

Adding fin to the bottom of the system in cases 1 and 3 increases the melting rate of the PCM at the base of the heat exchanger (the place where the solid PCM is collected due to the buoyancy effect). This behavior is caused by the direct and continuous adhesion between the based horizontal flat fin and the solid part of the PCM till the end of the melting process. This effect clearly appears with times over 1200 s (Figure 11). By comparing the figures of both cases 6 and 7 with cases of 1 and 3, a small portion of the solid-state appears at the base of the heat exchanger in cases 6 and 7, and the solid PCM totally disappears within 2400 s. On the other hand, the solid PCM is slightly bigger in cases 6 and 7 in the other regions. This is caused by the small thickness of the fins in those cases (the thickness reduces to compensation to adding the new fin at the base). The effect of the base fin also appears on the temperature profile, as shown in Figure 12. The figure shows that the temperature at the bottom of the exchange exchanger in cases 6 and 7 is slightly higher than those in cases 1 and 3, respectively. In the other regions, the temperature shows a higher value due to the small fins used in cases 6 and 7 as explained previously.

**Figure 13:**
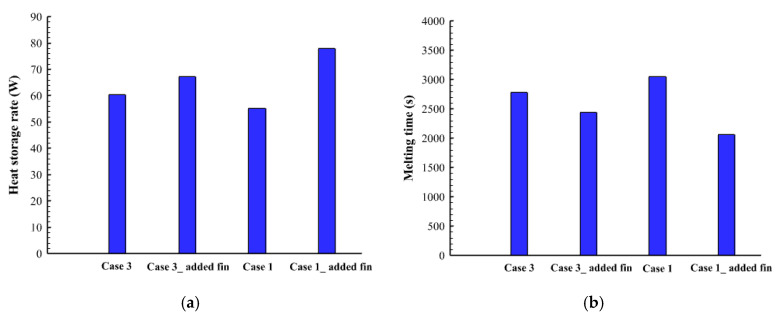
(**a**) Heat storage rate in Watt, (**b**) Melting time in seconds for cases 1, 3, 6, and 7 during the melting process.

Figure 13 shows the heat storage straight and the total melting time for cases 1, 3, 6, and 7. It clearly shows that case 6 presents the most efficient performance with a higher heat storage rate and lower melting time. This behavior is caused by the uniform size of the fins which can distribute the heat uniformly in the domain even the thickness is reduced. However, in case 7 the reduction of the fins thickness affects the heat distribution in the domain because of the non-uniform sizes of the fins, including a small fan at the top of the system.

**Figure 14:**
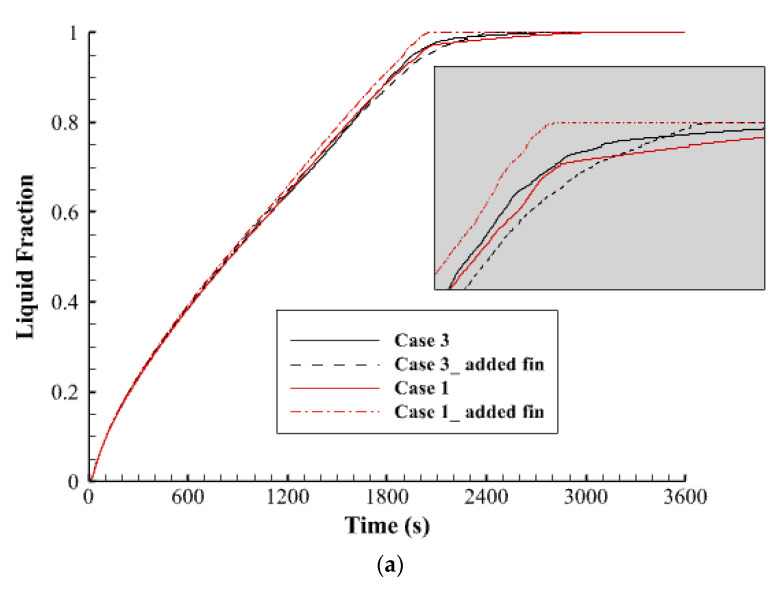

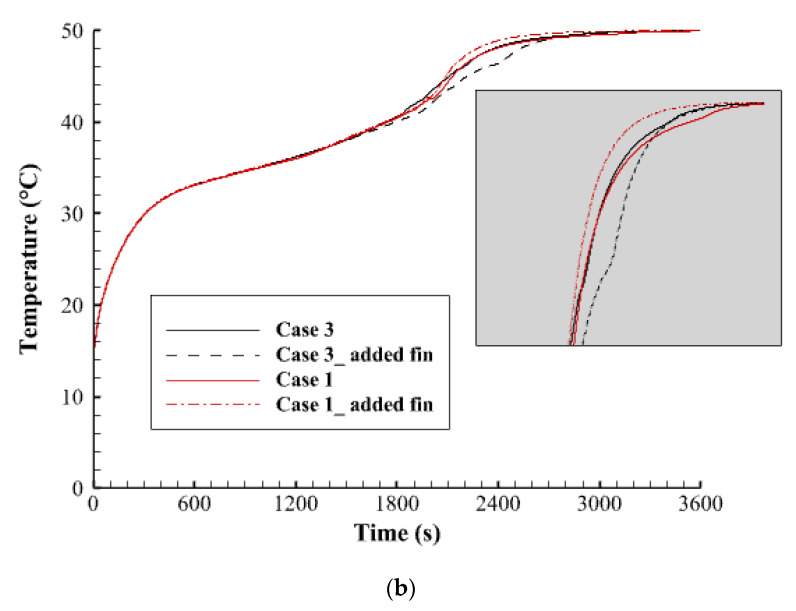
(**a**) Liquid fraction, (**b**) Average temperature, for cases 1, 3, 6, and 7 during the melting process.

Figure 14 shows the liquid fraction history and the temperature profile for cases 1, 3, 6, and 7 during the melting process. Figure 14a clearly indicates that the melting process of all the cases had the same pattern till the first 1800 s and more than 95% of the entire PCM is melted. Thereafter, the progress changes; the total PCM in case 6 melts faster than the other cases and shows a linear relationship, however, the other cases present a slower procedure. This behavior could be copied to the average temperature profile as illustrated in Figure 14b. The temperatures rise to 41 °C at the same time for all the cases at 1800 s. then the profiles take different patterns, and the temperature in case 6 reaches the equilibrium point faster than the other cases.

Table 6 shows the values of the heat storage rate and the melting time for cases 1, 3, 6, and 7. Case 6 was found as the best case with a 77.9 W with storage rate which is higher by 29%, 22.5%, and 13.5% over cases 1, 3, and 7, respectively. The melting time of case six (2057 s) is also, shorter than cases 1, 3, and 7 by 48.5%, 35.5%, and 18.6%, respectively.

## 6. Conclusions

This study was to examine the accelerated charging response of a latent TES system based on PCM confinement of triple-tube configuration with an internal circular fin array. The aim was to explore how adjusting the fin geometric dimensions and distribution patterns along the heat-flow direction allow superior heat transfer enhancement on the PCM side. To find the optimum fin structure, various fin arrangements were considered and compared in terms of melting front behavior, temperature distribution, melting completion time, and heat storage rate. All cases were maintained the same design limitations of fin material and volume usage. The major conclusions can be summarized as follows:

(1) Modifying the geometric dimensions of the fins and their distribution patterns along the heat-flow direction has a substantial impact on the melting enhancement potential of circular fins. Adjustment of the fin dimensions in different regions of the heat exchanger can save up to 10.4% of the melting completion time and improve the thermal storage rate by up to 9.3% compared to the reference case of uniform fin dimensions within the same fin volume limitations.
(2) Adding an extra fin at the base of the storage system affects the overall enhancement potential of circular fins. The fin array of non-uniform fin dimensions is generally more affected than the reference case of uniform fin dimensions because the fin structure of non-uniform fin dimensions promotes higher flow resistance to be generated, which, in turn, negatively affects the natural convection’s beneficial impact during the melting mode.
(3) Increasing the number of circular fins from five to six fins substantially improves the melting characteristics of PCM. However, a better melting rate can be achieved if the fin structure of uniform dimensions is applied. Compared to the case of nonuniform fin dimensions, the heat storage rate and the melting time can be improved by 13.5% and the melting completion time can be saved by up to 18.6%.

Eventually, the author suggests that for such cases, adding a flat fin to the base of the geometry improves the thermal performance obviously. This fact is clearly stated in Figure 6, which is considered as the optimum case in this work. Studying different sizes and angles for a pointed finned case could be considered in future work to study the slipping effect of the PCM on the sloped sides of the fin.

## Figures and Tables

**Table 1:** The dimensions of circular fins in millimeters for all the proposed cases.

	Fin 1 (Lowest Fin)	Fin 2	Fin 3	Fin 4	Fin 5	Added Fin to the Bottom
Case 0	-	-	-	-	-	-
Case 1	2 × 5	2 × 5	2 × 5	2 × 5	2 × 5	-
Case 2	2 × 9	2 × 7	2 × 5	2 × 3	2 × 1	-
Case 3	2 × 7	2 × 6	2 × 5	2 × 4	2 × 3	-
Case 4	3 × 5	2.5 × 5	2 × 5	1.5 × 5	1 × 5	-
Case 5	2.5 × 5	2.25 × 5	2 × 5	1.75 × 5	1.5 × 5	-
Case 6	1.42 × 7	1.42 × 6	1.42 × 5	1.42 × 4	1.42 × 3	1.42 × 20

**Table 2:** Thermodynamic properties of the PCM used [44].

**Properties**	$\rho l\;[\mathrm{kg}/m^{3}]$	$\rho s\;[\mathrm{kg}/m^{3}]$	**L_f_ [kJ/kg]**	**C_p_ [kJ/kg.K]**	**K [W/m.K]**	**µ [N.s/m^2^]**	**T_L_ [°C]**	**T_S_ [°C]**	**β [J/K]**
Values	770	860	170	2	0.2	0.023	36	29	0.0006

**Table 3:** Effect of cell number and time step size on the melting time.

Number of Cells	28,500	43,000			81,620
Time step size (s)	0.2	0.1	0.2	0.4	0.2
Melting time	4644	4733	4727	4701	4739

**Table 4:** Heat storage rate and the total melting time for case 1 and the finless case.

Studied Model	Heat Storage Rate (W)	Melting Time (s)
Case 1	55.1	3056
No-fin	36.2	4654

**Table 5:** Heat storage rate and the total melting time for different cases of the non-uniform fin sizes during the melting process.

Studied Model	Heat Storage Rate	Melting Time
Case 2	57.2	2929
Case 3	60.3	2787
Case 4	55.9	3014
Case 5	54.7	3077

**Table 6:** Heat storage rate and the total melting time for cases 1, 3, 6, and 7 during the melting process.

Studied Model	Heat Storage Rate	Melting Time
Case 1	55.1	3056
Case 6	77.9	2057
Case 3	60.3	2787
Case 7	67.3	2440

## Data Availability

Not applicable.

## Nomenclature

** $A_{m}$ **	The mushy zone constant
*C*	Inertial coefficient
$C_{p}$	PCM specific heat (J/kgK)
*D*	Hydrolic diameter (m)
g	Gravitational acceleration (m/s^2^)
K	thermal conductivity (W/mK)
L	Latent heat of fusion (J/kg)
m	PCM mass (kg)
P	Pressure (Pa)
$\dot{Q}$	Solidification rate (J)
$t_{m}$	Solidification time (s)
T	Temperature (K)
$T_{m}$	Melting point temperature (K)
$u_{i}$	Velocity component (m/s)
$\vec{V}$	Velocity vector (m/s)
**Greek Symbols**	
β	Thermal expansion coefficient (1/K)
λ	Liquid fraction
α	Thermal diffusivity (m^2^/s)
μ	Dynamic viscosity (kg/ms)
ρ	Density (kg/m^3^)
